# An approach for setting evidence-based and stakeholder-informed research priorities in low- and middle-income countries

**DOI:** 10.2471/BLT.15.162966

**Published:** 2015-02-12

**Authors:** Eva A Rehfuess, Solange Durão, Patrick Kyamanywa, Joerg J Meerpohl, Taryn Young, Anke Rohwer, on behalf of the CEBHA+, consortium

**Affiliations:** a Institute for Medical Informatics, Biometry and Epidemiology, LMU Munich, Marchioninistr. 15, 81377 Munich, Germany.; b Cochrane South Africa, South African Medical Research Council, Cape Town, South Africa.; c University of Rwanda, Butare, Rwanda.; d Cochrane Germany, Freiburg, Germany.; e Centre for Evidence-based Health Care, Stellenbosch University, Cape Town, South Africa.

## Abstract

To derive evidence-based and stakeholder-informed research priorities for implementation in African settings, the international research consortium Collaboration for Evidence-Based Healthcare and Public Health in Africa (CEBHA+) developed and applied a pragmatic approach. First, an online survey and face-to-face consultation between CEBHA+ partners and policy-makers generated priority research areas. Second, evidence maps for these priority research areas identified gaps and related priority research questions. Finally, study protocols were developed for inclusion within a grant proposal. Policy and practice representatives were involved throughout the process. Tuberculosis, diabetes, hypertension and road traffic injuries were selected as priority research areas. Evidence maps covered screening and models of care for diabetes and hypertension, population-level prevention of diabetes and hypertension and their risk factors, and prevention and management of road traffic injuries. Analysis of these maps yielded three priority research questions on hypertension and diabetes and one on road traffic injuries. The four resulting study protocols employ a broad range of primary and secondary research methods; a fifth promotes an integrated methodological approach across all research activities. The CEBHA+ approach, in particular evidence mapping, helped to formulate research questions and study protocols that would be owned by African partners, fill gaps in the evidence base, address policy and practice needs and be feasible given the existing research infrastructure and expertise. The consortium believes that the continuous involvement of decision-makers throughout the research process is an important means of ensuring that studies are relevant to the African context and that findings are rapidly implemented.

## ^Résumé^

Afin de déterminer, à partir d'éléments factuels et avec l'apport des parties prenantes, les priorités de recherche pouvant être mises en œuvre dans les pays d'Afrique, le consortium de recherche international *Collaboration for Evidence-Based Health Care and Public Health in Africa* (CEBHA+) a élaboré et appliqué une démarche pragmatique. Tout d'abord, une enquête en ligne et une consultation en face à face entre les partenaires du CEBHA+ et les responsables politiques ont permis de mettre en lumière les domaines de recherche prioritaires. Ensuite, des listes documentaires relatives à ces domaines de recherche prioritaires ont permis d'identifier les lacunes ainsi que des questions connexes prioritaires en matière de recherche. Enfin, des protocoles d'étude ont été mis au point en vue d'être intégrés à une proposition de subvention. Des représentants de la mise en œuvre des politiques ont participé à l'ensemble du processus. La tuberculose, le diabète, l'hypertension et les blessures de la route ont été sélectionnés comme domaines de recherche prioritaires. Les listes documentaires portaient sur le dépistage et les modèles de soins du diabète et de l'hypertension, sur la prévention de ces maladies ainsi que sur leurs facteurs de risque au niveau de la population, et sur la prévention et la prise en charge des blessures de la route. L'analyse de ces listes a permis de dégager trois questions de recherche prioritaires portant sur l'hypertension et le diabète et une sur les blessures de la route. Les quatre protocoles d'étude en découlant utilisent toute une série de méthodes de recherche primaire et secondaire; un cinquième favorise une démarche méthodologique intégrée sur l'ensemble des activités de recherche. La démarche du CEBHA+, en particulier la constitution de listes documentaires, a permis de formuler les questions de recherche et les protocoles d'étude qui reviendront aux partenaires africains, de combler les lacunes des bases de données et de répondre aux besoins en matière de politiques et de pratiques; elle s'est également révélée applicable compte tenu de l'expertise et de l'infrastructure de recherche existantes. Pour le consortium, l'implication continue des décisionnaires dans le processus de recherche est un important moyen de garantir que les études soient pertinentes pour les pays africains et que leurs résultats soient rapidement mis en œuvre.

## Resumen

Para obtener las prioridades de investigación documentadas y comunicadas a las partes interesadas para su implementación en África, el consorcio de investigación internacional Asociación de Asistencia Médica Basada en Evidencia en África (CEBHA+, por sus siglas en inglés), desarrolló y aplicó un enfoque pragmático. En primer lugar, una encuesta en línea y una consulta presencial entre socios de la CEBHA+ y responsables políticos originó los sectores de investigación prioritarios. En segundo lugar, unos mapas documentados para estos sectores de investigación prioritarios identificaron disparidades y asuntos relativos a la investigación prioritaria. Por último, se desarrollaron protocolos de estudio para incluirlos dentro de una propuesta de subvención. Los representantes políticos y prácticos participaron durante todo el proceso. Los sectores de investigación prioritarios seleccionados fueron la tuberculosis, la diabetes, la hipertensión y los traumatismos provocados por accidentes de tráfico. Los mapas documentados abordaron el examen y los modelos de asistencia de la diabetes y la hipertensión, la prevención de la diabetes y la hipertensión a nivel de población, sus factores de riesgo y la prevención y gestión de traumatismos provocados por accidentes de tráfico. Los análisis de estos mapas generaron tres asuntos de investigación de prioridades sobre hipertensión y diabetes y uno sobre traumatismos provocados por accidentes de tráfico. Los cuatro protocolos de estudio resultantes emplean una amplia gama de métodos de investigación primarios y secundarios; un quinto presenta un enfoque metodológico integrado a través de todas las actividades de investigación. El enfoque de la CEBHA+, concretamente los mapas documentados, contribuyó a formular cuestiones y protocolos de estudio sobre la investigación que pertenecerían a socios africanos, subsanarían diferencias en la base de pruebas, abordarían las necesidades sobre políticas y prácticas y serían viables gracias a la infraestructura y experiencia de investigación existentes. El consorcio considera que la constante implicación de los responsables de la toma de decisiones a lo largo del proceso de investigación es un medio importante para garantizar que los estudios se correspondan al contexto africano y que los resultados se implementen con rapidez.

## ملخص

حرصًا على اشتقاق أولويات البحوث القائمة على الأدلة والمرتكزة على الجهات المعنية لتنفيذها في البلدان الأفريقية، فقد ساهمت الجهود التعاونية للرعاية الصحية القائمة على الأدلة والصحة العامة في أفريقيا (CEBHA+‎) من جانب اتحاد البحوث الدولية في إعداد وتطبيق أسلوب منهجي عملي. أولاً، تم من خلال استطلاع للرأي عبر الإنترنت والمشورة المباشرة وجهًا لوجه ما بين شركاء الجهود التعاونية للرعاية الصحية القائمة على الأدلة وصناع القرار إنتاج المجالات البحثية ذات الأولوية. ثانيًا، قامت خرائط الأدلة لهذه المجالات البحثية ذات الأولوية بتحديد الثغرات والأسئلة المتعلقة بالبحوث ذات الأولوية. وأخيرًا، تم إعداد بروتوكولات الدراسة لتضمينها في المنحة المقترحة. كما شارك ممثلو السياسات والممارسات خلال جميع مراحل العملية. وتم اختيار الإصابات الناتجة عن أمراض السل، والسكري، وارتفاع ضغط الدم، والإصابات المرورية على الطرق كمجالات للبحوث ذات الأولوية. شملت خرائط الأدلة الفحوصات ونماذج الرعاية لمرض السكري وارتفاع ضغط الدم، والوقاية على مستوى السكان من مرض السكري وارتفاع ضغط الدم وعوامل المخاطرة المرتبطة بها، ومنع وقوع الإصابات المرورية على الطرق والتحكم فيها. أسفر تحليل هذه الخرائط عن ثلاثة أسئلة متعلقة بالبحوث ذات الأولوية حول ارتفاع ضغط الدم ومرض السكري وسؤال واحد عن الإصابات المرورية على الطرق. تستخدم أربعة من بروتوكولات الدراسة الناتجة مجموعة واسعة من أساليب البحوث الأساسية والثانوية؛ كما يقوم البروتوكول الخامس بتعزيز اتباع أسلوب منهجي متكامل عبر جميع الأنشطة البحثية. ساهم النهج المتبع من جانب الجهود التعاونية للرعاية الصحية القائمة على الأدلة، ولا سيما ترسيم الأدلة، في صياغة الأسئلة البحثية وبروتوكولات الدراسة التي يمتلكها الشركاء الأفارقة، وملء الثغرات في قاعدة الأدلة، وتلبية احتياجات السياسات والممارسات، وأن يكون بالإمكان تحقيقه نظرًا للبنية التحتية البحثية والخبرة الموجودة بالفعل. ويرى الاتحاد أن المشاركة المستمرة من صناع القرار في جميع مراحل عملية البحث هي وسيلة هامة لضمان تنفيذ الدراسات المرتبطة بالبيئة الأفريقية على جناح السرعة.

## 摘要

为了获知在非洲环境中实施循证以及利益相关者知情的研究重点，国际研究联合会非洲循证医疗保健和公共健康合作 (CEBHA+) 开发并运用实用方法。首先，根据 CEBHA+ 合作伙伴和决策者之间的在线调查和面对面咨询，产生重点研究领域。其次，这些重点研究领域的证据映射确定差距和相关的重点研究问题。最后，制定研究方案，纳入拨款提案中。整个过程包括政策和实践代表。选择结核病、糖尿病、高血压和道路交通伤害为重点研究领域。证据映射涵盖糖尿病和高血压的筛查和护理模式，全民预防糖尿病和高血压及其风险因素，以及道路交通伤害的预防和管理。这些映射的分析可产生三个有关高血压和糖尿病及一个有关道路交通伤害的重点研究问题。四个结果调查草案运用广泛的主要和次要研究方法；第五个提倡使用所有研究活动的综合方法对策。CEBHA+ 方法，尤其是证据映射，有助于制定将为非洲合作伙伴所有的研究问题和调查草案，弥补循证的差距，满足政策和实践需求，在现有研究基础设施和专业知识基础上切实可行。联盟认为决策者持续参与整个研究过程是确保研究符合非洲情况和快速实施调查结果的重要方式。

## Резюме

Чтобы определить приоритетные направления исследований, которые основываются на имеющихся данных и о которых сообщается заинтересованным сторонам, для реализации в условиях Африки, международным исследовательским консорциумом «Сотрудничество для реализации научно обоснованной медико-санитарной помощи и общественного здравоохранения в Африке» (CEBHA+) был разработан и применен прагматический подход. Вначале с помощью электронного опроса мнений и консультирования в ходе личных встреч между партнерами CEBHA+ и лицами, формирующими политику, были определены приоритетные области исследований. Затем путем составления карт имеющихся данных для этих приоритетных областей исследований были выявлены пробелы и поставлены соответствующие исследовательские вопросы. В заключение были разработаны протоколы исследования для включения в заявку на грант. Представители сторон, отвечающих как за формирование политики, так и за ее реализацию на практике, принимали участие во всех этапах процесса. В качестве приоритетных направлений исследований были выбраны туберкулез, сахарный диабет, гипертония и телесные повреждения в результате дорожно-транспортного происшествия. Карты имеющихся данных содержали данные скрининговых обследований и модели лечения диабета и гипертонии, данные о мерах по профилактике сахарного диабета и гипертонии на уровне населения и факторы риска этих состояний, а также данные о мерах по профилактике и снижению количества телесных повреждений, полученных в результате дорожно-транспортного происшествия. В результате анализа этих карт были поставлены три исследовательских вопроса для приоритетных направлений гипертонии и сахарного диабета и один — для телесных повреждений в результате дорожно-транспортного происшествия. Четыре разработанных протокола исследования содержат разнообразные методы первичного и вторичного исследования, и в пятом предложен комплексный методологический подход, охватывающий всю исследовательскую деятельность. Благодаря подходу CEBHA+, в частности благодаря составлению карт имеющихся данных, удалось сформулировать исследовательские вопросы и составить протоколы исследования, которые были бы переданы африканским партнерам. Также благодаря этому были заполнены пробелы в фактологической базе, устранены проблемы в области формирования политики и практики и удалось обеспечить целесообразность в условиях существующей исследовательской инфраструктуры и имеющихся специальных знаний и опыта. Консорциум считает важным непрерывное участие лиц, принимающих решения, во всех этапах исследования, поскольку это гарантирует, что исследования целесообразны для проведения в условиях Африки, а их результаты быстро начинают учитываться на практике.

## Introduction

Mortality in sub-Saharan Africa is still predominantly caused by human immunodeficiency virus/acquired immunodeficiency syndrome (HIV/AIDS), malaria and other infectious diseases. However, premature deaths due to noncommunicable diseases and unintentional injuries are increasing.^1^ Furthermore, Africa is facing significant challenges in the provision of preventative and curative health care. This is the result of a combination of factors – including insufficient human resources, poor health system infrastructure, limited supplies of essential medication and technology and suboptimal health-care seeking.^2^^–^^4^

While there has been a significant increase in health research conducted in the region in recent years,^5^ the overall research has not been commensurate with the challenges in terms of quantity or quality.^6^ Much of the research undertaken is less informative than it should be, often because of a mismatch between research required by decision-makers and that conducted by academic institutions. In some instances, the research agenda is driven by funders (including industry) and thus concerned with international rather than national or local problems. Furthermore, usability of findings tends to be hampered by limitations in quality of conduct, analysis and reporting of studies. Thus there is a need in the research field “to increase value and to reduce waste”,^7^^–^^9^ especially in resource-constrained settings such as Africa.

Evidence-based approaches to address health problems are recognized as best practice. Evidence-based public health draws on the principles of evidence-based health care^10^ and is defined as the *“*integration of the best available evidence with the knowledge and considered judgments from stakeholders and experts to benefit the needs of a population”.^11^

When allocating resources, policy-makers and health-care practitioners need to consider the significance of the health problem; the potential benefits and harms of the intervention and the quality of evidence on effectiveness. The cost and cost–effectiveness must also be weighed up, along with personal values and preferences, feasibility, acceptability and equity. To achieve evidence-based decision-making, data from rigorous primary research and evidence syntheses relevant to the African context must expand and translation of evidence into policy and practice must be enhanced.^12^^,^^13^

The Collaboration for Evidence-Based Healthcare and Public Health in Africa (CEBHA+) emerged from the Collaboration for Evidence Based Healthcare in Africa (www.cebha.org). CEBHA+ promotes evidence-based health care principles through (i) identifying relevant and context-sensitive research priorities; (ii) conducting robust, internationally competitive research; and (iii) linking primary research with evidence synthesis, implementation research, policy and practice.

Currently, the consortium comprises eight African partners in five countries (Ethiopia, Malawi, Rwanda, South Africa and Uganda), two German partners and two associate partners. As part of the preparatory phase, the consortium developed a pragmatic approach for setting evidence-based and stakeholder-informed research priorities to ensure that the research would be: (i) unique – to avoid unnecessary duplication and fill a gap in the African and/or international evidence base; (ii) relevant – to address pressing questions asked by African decision-makers; (iii) context-sensitive – to facilitate usability in African settings; (iv) feasible – to ensure that research can be conducted with existing interest, expertise and resources; and (v) high quality – to minimize limitations in quality of conduct, analysis and reporting of studies. This paper describes the development and application of this approach and discusses its strengths and limitations.

## Developing research priorities

We followed a three-step participatory process. Representatives of the policy and practice community were involved throughout, as continuous interaction can help identify challenges in need of solutions and increase the chances of research findings being translated into policy.

### Step 1

Through an online survey and face-to-face consultations we developed a list of priority research areas. To do so, we carried out an online survey with all African partners and African policy-makers in the participating countries, with the latter selected to reflect existing interactions between research and practice in each country. Both groups were asked to complete the survey from an institutional perspective, having consulted with colleagues through individual interactions or round table discussions. The survey aimed to assess potential priority research areas drawing on the international evidence base as well as the expertise and interests of participating institutions. It was structured in four sections: (i) priority diseases, drawing on but not limited to the 25 most important diseases in sub-Saharan Africa based on estimated disability-adjusted life years (DALYs);^1^ (ii) the 25 most important risk factors in sub-Saharan Africa also based on estimated DALYS;^14^ (iii) priority interventions against diseases and risk factors; and (iv) ongoing projects by partners. We obtained a waiver from the Ethics Committee of the LMU Munich, Germany, given the low-risk nature of the survey. All data were handled anonymously. The survey was conducted in March and early April 2014 using Survey Monkey (https://www.surveymonkey.com/). Survey data were analysed descriptively.

An initial shortlist of priority research areas derived from the online survey provided the starting point for face-to-face consultations during a three-day meeting in Addis Ababa, Ethiopia, in April 2014. Participants included one or more representatives of all partners and high-level health policy-makers from Rwanda, South Africa and Uganda. A two-stage interactive group process was followed to achieve consensus, with participants from a given country initially selecting their first choice, a subsequent grouping of priority research areas and in-depth discussions regarding those selected by at least three countries. With reference to existing checklists,^15^^,^^16^ participants were asked to consider four criteria in prioritizing: (i) magnitude or seriousness of the health problem; (ii) research and other strengths of the consortium in the respective area; (iii) requirements by the funder and related strategic advantages and/or disadvantages; and (iv) feasibility of achieving meaningful results given available resources and timelines.

### Step 2

Through evidence maps, we identified priority research questions that would fill a gap in the African evidence base. These evidence maps provided an overview of the existing evidence for the priority research areas from step 1. Expanding on previous work,^17^ we developed methodological guidance comprising seven steps: developing a framework, formulating a clear question, defining criteria for inclusion of studies, conducting systematic searches, selecting studies for inclusion, extracting data and presenting results (Table 1). Importantly, evidence maps focused on systematic reviews. Depending on the question and resources permitting, primary studies and/or guidelines were also considered.

**Table 1:** Developing an evidence map in seven steps

Step	Description	Example
1. Developing a framework	Describe broad research area and/or use logic model to illustrate framework, using published logic model templates^18^	Comprehensive models of care for diabetes and hypertension
2. Formulating a clear question	Formulate broad question using the PICO format	What are the effects of comprehensive service delivery models for management of chronic diseases (with a focus on diabetes and hypertension) in adults, across the whole spectrum of prevention, early diagnosis and treatment?
3. Defining criteria for inclusion of studies	Develop criteria related to population, intervention/indicator and study designs Do not use criteria related to comparisons or outcomes	Participants: Adults (> 18 years), excluding pregnant women Interventions: Any comprehensive model of service delivery or model of care, addressing prevention, early diagnosis or treatment of diabetes and/or hypertension; or a combination of these Studies: systematic reviews, defined as those that had predetermined objectives, predetermined criteria for eligibility, searched at least two data sources, of which one was an electronic database, and performed data extraction and risk of bias assessment. We also considered randomized controlled trials in case of finding a limited number of systematic reviews.
4. Conducting systematic searches	Pre-specify a search strategy focusing on population and intervention Search for published and unpublished systematic reviews in the following systematic review and health research databases Cochrane database (www.cochranelibrary.com) Health Evidence (www.healthevidence.org) EPPI Centre database (http://eppi.ioe.ac.uk/cms) 3ie database (www.3ieimpact.org/evidence/) Prospero (ongoing systematic reviews) (www.crd.york.ac.uk/PROSPERO) PubMed (www.ncbi.nlm.nih.gov/pubmed) Embase (www.elsevier.com/online-tools/embase) AfricaBib databases (in particular Africana Periodical Literature and African Women) (www.africabib.org) WHO's Global Health Library (www.globalhealthlibrary.net) TRIP database (www.tripdatabase.com) Consider searching other relevant databases, as needed Time and resources permitting, subsequently conduct searches for primary studies and/or guidelines, with the most important guideline databases being GIN database (www.g-i-n.net/library/international-guidelines-library) National guideline clearinghouse (USA) (www.guideline.gov)	A combination of search terms related to delivery of health care, diabetes, hypertension and systematic reviews was used and the search string adapted to each database. Specific search strategies are reported for each database.
5. Selecting studies for inclusion	Select studies for inclusion by first screening titles and abstracts for potentially eligible studies Conduct full text screening of potentially eligible studies	One author screened all the titles and abstracts of the search outputs to discard the citations that were not relevant to the question. Both authors then did a second round of screening to identify potentially eligible studies. Full text screening of seemingly relevant studies was done by two authors independently.
6. Extracting data	Pre-specify data extraction form, which should include citation details, characteristics of the systematic review, primary study or guideline, characteristics of the population, intervention and comparisons, primary and secondary outcomes and quantitative or qualitative results Extract relevant data onto data extraction form	One author extracted data of the included systematic reviews onto a form containing: Study ID and citation Included study designs Geographical details Number of included studies and participants Characteristics of populations Characteristics of interventions and comparisons Reported outcomes Main results
7. Presenting results	Present findings descriptively in table format and, where appropriate, through a visual mapping of the intervention according to intervention type and outcome Note that evidence maps do not comprise risk of bias assessment or formal evidence synthesis	Results for each of the included systematic reviews were presented in table format in relation to each of the six intervention categories identified. We did not assess the quality of the systematic reviews. The following example relates to one of the included systematic reviews: Study ID and title: Smith 2009 – Private local pharmacies in low-and middle-income countries: a review of interventions to enhance their role in public health No. of included studies (participants): 18 studies overall, 2 studies (60) related to hypertension Types of included studies: Before-after, crossover design Location of included studies: Nigeria Participants: Hypertensive patients on anti-hypertensive medication reporting to a local, private pharmacy Interventions: Pharmaceutical care intervention: Information and advice to individual patients Monthly goal-directed counselling Comparisons: Usual care Outcomes: Blood pressure Quality of life Main results: Significant reductions in blood pressure

PICO: population intervention/indicator comparison outcome; WHO: World Health Organization.

Subsequently, we identified gaps in the evidence base and formulated research questions to fill these gaps. This involved discussion between researchers and decision-makers at the national or provincial level to ensure that the research to be conducted would be able to answer a policy-relevant question and to decide on the most appropriate way to do so. In addition, researchers involved in relevant activities were consulted to check that priority research questions would build on existing research and not duplicate current research by other groups. Between June and October 2014, evidence maps were created by cross-national research teams with methodological support from the LMU Munich.

### Step 3

We developed study protocols towards a full grant proposal. The cross-national research teams engaged with policy-makers to jointly develop protocols using email, voice calls and a two-day face-to-face meeting. Depending on the specific content and methodological expertise required, we involved additional scientists within partner institutions or recruited additional partner institutions. Study protocols were developed between September and December 2014.

## Identified research priorities

### Priority research areas

The online survey was completed by seven out of eight partner institutions in six countries (Burundi, Ethiopia, Malawi, Rwanda, South Africa and Uganda) and by policy-makers in Malawi, Rwanda, South Africa and Uganda.

**Fig. 1:**
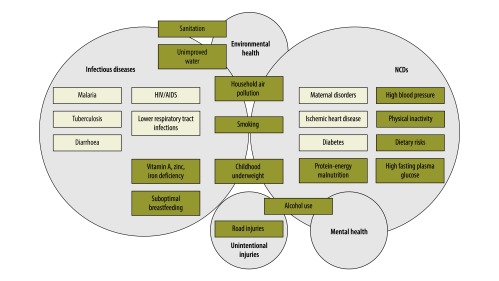
Priority research areas, diseases and risk factors as identified through the survey with African policy-makers and CEBHA+ partners

AIDS: acquired immunodeficiency syndrome; CEBHA+: Collaboration for Evidence-Based Healthcare and Public Health in Africa; HIV: human immunodeficiency virus; NCDs: noncommunicable diseases.

Note: The grey circles represent the priority research areas that were identified from the survey. The size of the circles indicates the importance of the respective research area as ranked by survey respondents. The light green boxes within the grey circles illustrate the priority diseases that were identified within the priority research areas. The dark green boxes illustrate the priority risk factors identified and associated with the priority diseases.

Both partners and decision-makers identified infectious diseases and noncommunicable diseases as the two most important problems but differed in their ranking of mental health, environmental health and unintentional injuries. At least three countries selected malaria, HIV/AIDS, lower respiratory tract infections, diarrhoeal diseases, protein-energy malnutrition, road traffic injuries, tuberculosis, maternal disorders and diabetes as priority problems for CEBHA+ (Fig. 1). At least three countries listed childhood underweight, suboptimal breastfeeding, high blood pressure, dietary risks, sanitation, high-fasting plasma glucose, unimproved water and physical inactivity as priority risk factors (Fig. 1). They prioritized population-level (i.e. primary prevention, secondary prevention, health systems and health policy interventions) over individual-level interventions (i.e. individual-level health care and tertiary prevention).

Following face-to-face consultation on these findings, partners selected tuberculosis, diabetes, hypertension and road traffic injuries as the priority research areas to focus on within the consortium. Despite their importance in terms of disease burden,^1^^,^^14^ mental health and environmental health topics were not selected, primarily because of insufficient expertise within the consortium to undertake high-quality research. There was consensus that all research activities required a population and/or health systems perspective and that each research activity would need to be taken forward jointly by at least three partner institutions. It was agreed that the research should be led and owned by African partners rather than by European collaborators or funding bodies.

### Priority research questions

Two evidence maps on diabetes and hypertension and one on road traffic injuries were developed; an evidence map on tuberculosis–HIV was initiated but not completed.

#### Evidence map 1

We reviewed the effects of comprehensive service delivery models for the management of diabetes and hypertension in adults across the whole spectrum of prevention, early diagnosis and treatment. Eligible outcomes were incidence of diabetes and hypertension, adherence to care, number and severity of complications, avoidable hospital admissions and mortality. Searches retrieved 5516 records, with 55 full texts screened. Twenty-four articles were included, reporting on 16 systematic reviews. These addressed interventions delivered by pharmacists (four reviews), interventions delivered by nurses, community health workers and other non-physician health-care workers (three reviews), screening interventions (three reviews), disease and care management interventions (two reviews), health system and organization of care interventions (two reviews) and multifaceted interventions (e.g. combining educational, provider roles, organizational interventions; two reviews). No systematic review addressed integrated models of care for diabetes or hypertension. Most systematic reviews included studies in high-income settings, with only two systematic reviews focusing on studies in low- and middle-income countries. Based on the identified evidence gaps, we formulated questions on the effectiveness of screening approaches and integrated models of care for diabetes and hypertension in sub-Saharan Africa.

#### Evidence map 2

We reviewed the effects of population-level interventions for preventing diabetes and hypertension. Eligible interventions comprised policies, regulations and environmental changes addressing risk factors for diabetes and hypertension, such as unhealthy diets and excessive body weight. We considered outcomes related to process (e.g. coverage), behaviour (e.g. physical activity, nutritional intake) and health (e.g. cardiovascular morbidity and mortality). Due to time constraints, only 2976 of 5528 records identified through searches were screened, with 82 full texts assessed and 14 systematic reviews included. These covered workplace (three reviews), school (five reviews) and community or population-based interventions (six reviews). Most reviews focused on evidence from high-income settings, reporting on widely differing types of interventions and outcomes; many did not report synthesized results. Based on the analysis of the existing evidence, a question on the effectiveness of population-level interventions to prevent diabetes and hypertension in sub-Saharan Africa was formulated.

#### Evidence map 3

We reviewed the effects of interventions for the prevention and response to road traffic injuries addressing road users, vehicles, physical road environments and legislation or care protocols. Outcomes of interest were hospital admissions and mortality attributable to road traffic injuries. Both systematic reviews and randomized controlled trials were considered. Systematic searches retrieved 968 records, yielding 15 eligible studies. Using the reference lists of included studies, an additional 11 eligible records were retrieved, yielding a total of 26 studies. Most concentrated on the effectiveness of interventions to reduce the occurrence of road traffic crashes, i.e. education and training, licencing, alcohol restriction and enforcement of alcohol limits, visibility enhancement for road users, street lighting and visibility aids, enforcement of speed limits, bicycle helmet and booster seat legislation. Only two studies were concerned with the response by ambulance and hospital staff after the crash. Except for South Africa, the systematic reviews only included data from high-income countries; randomized controlled trials were all from high-income countries. Thus a need to strengthen the evidence base regarding the implementation of road traffic injury prevention in sub-Saharan Africa was recognized.

### Study protocols

Four study protocols were developed to address identified priority research questions; a fifth promoted a rigorous methodological approach across all research activities: (i) evidence-informed policies and practices on screening approaches for hypertension and diabetes, and those at high risk of cardiovascular disease in sub-Saharan Africa (Ethiopia, Malawi, Rwanda, South Africa); (ii) evidence-informed policies and practices on integrated models of health care delivery for hypertension and diabetes in sub-Saharan Africa (Ethiopia, Malawi, Rwanda, South Africa); (iii) evidence-informed policies and practices on population-level interventions to prevent diabetes and hypertension in sub-Saharan Africa (Malawi, Rwanda, South Africa); (iv) improved implementation of road traffic injury prevention interventions in sub-Saharan Africa (Rwanda, South Africa, Uganda); and (v) promotion of an integrated, rigorous methodological approach across research tasks and components (all five countries).

**Fig. 2:**
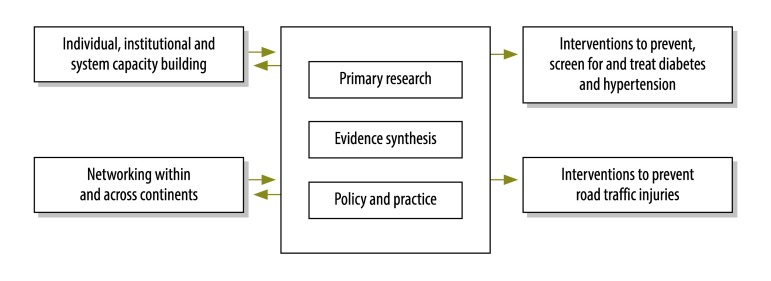
Overview of CEBHA+ research and implementation framework as applied to diabetes, hypertension and road traffic injuries

Each protocol represents a full research package, where different sub-questions are addressed using a range of methods, including situation analysis, diagnostic studies, observational epidemiology, intervention effectiveness, qualitative research and process evaluation, as well as systematic reviews, overviews of systematic reviews, guidelines and evidence-informed policy briefs. As shown in Fig. 2, all five protocols are embedded within the CEBHA+ research and implementation framework that intends to link primary research, evidence synthesis and implementation with policy and practice. The protocols are complemented by and integrated with activities on capacity-building and networking aiming to develop knowledge and skills, long-term infrastructure and research-to-policy collaborations.

CEBHA+: Collaboration for Evidence-Based Healthcare and Public Health in Africa.

## Discussion

To identify priority research areas and questions relevant for the African context, we developed and applied a structured participatory approach. This approach connects the international evidence base with the needs of policy-makers and the expertise and interests of researchers. Major evidence gaps and research needs were highlighted regarding prevention and integrated treatment of hypertension and diabetes and prevention of road traffic injuries in sub-Saharan Africa. Five study protocols – four on priority research questions and one on accompanying methods – were developed and included in a grant proposal for a five-year implementation phase between 2016 and 2020, which the German Federal Ministry of Education and Research has decided to fund.^19^ Notably, this pragmatic approach for deriving research priorities for an international research consortium can be applied rapidly, even in low- and middle-income settings.

### Strengths and limitations

Health research priority-setting is conducted to identify research with the greatest potential health and societal benefits. A general framework for setting priorities in health research does not exist;^15^^,^^20^^,^^21^ to date such undertakings are very heterogeneous in terms of scope and target audience as well as methods employed. Indeed, the optimal approach depends on the needs of a given exercise,^15^ with methods selected based on context, time and resource constraints.^20^

A major strength of our approach is that it is grounded in evidence, both as a starting point for the initial list of priority research areas in step 1 and as a means of identifying specific research questions in step 2. Evidence maps as a means of assessing the evidence base in a relatively quick way are the most novel feature of the approach. A comprehensive assessment of the current evidence base is often lacking in research priority-setting exercises, with these usually making use of simple literature reviews or expert consultation.^15^^,^^20^

The product of evidence mapping is, however, not necessarily comprehensive, especially where searches are limited to systematic reviews. Indeed, for several of the priority research questions CEBHA+ partners thought it was necessary to conduct a more comprehensive and thorough but more time-consuming overview of systematic reviews or to undertake systematic reviews of sub-questions as part of the full proposal. Evidence maps can be developed more rapidly than systematic reviews. Nevertheless, the process tends to take two to three months and requires a dedicated research team with expertise in undertaking searches, screening records and extracting and interpreting data. Due to limited time and lack of personnel, there was incomplete screening of the search results for the evidence maps on population-level interventions to prevent diabetes and hypertension. Also, a fourth evidence map on the implementation of tuberculosis-HIV interventions was initiated but not completed; consequently, no research task was developed for infectious diseases.

Our guide to evidence maps could be adapted to derive research priorities for different audiences and purposes in the African setting and beyond. It could be applied to any area of health research at any level, whether local, national, regional or international. We learnt, however, that it cannot be taken for granted that this guide is self-explanatory; instead, its rigorous application requires specific, ideally hands-on, training.

We employed a combination of metric- and consensus-based approaches to derive priority research areas. A weakness of our approach is that, due to time and resource constraints, we did not utilize a formal method for building consensus, such as a Delphi or nominal group technique. In view of the purpose of our exercise, i.e. for an international research consortium to develop a joint grant proposal, the selection of priority research questions was based on analysis of the evidence maps and discussion with decision-makers. Planning for implementation is inherent in our approach.^15^ Very few priority-setting exercises systematically assess whether the research priorities generated have any impact.^20^ While we will only be able to evaluate impact on research and policy and practice in a few years’ time, the fact that our proposal secured a large grant can be considered an intermediate indicator of success.

There was continuous involvement of relevant decision-makers throughout the research process from identification of the question and proposal development through to study conduct, publication and use of results. This was necessary to develop research questions that would address policy and practice needs and that would be achievable given existing resources. This involvement should facilitate a more rapid uptake of research results in policy and practice, although whether this is achieved will need to be evaluated carefully. Involving a broad range of stakeholders is considered an important feature of valid research priority-setting.^20^^,^^22^^,^^23^ Partners felt strongly that research must be owned by African partners, and that having Africans choose their own research priorities is an important means to achieve this goal.

## Conclusion

The pragmatic approach outlined here facilitates research that is unique, relevant, context-sensitive, feasible and of high-quality in the context of an international research consortium. Our approach to setting evidence-based and stakeholder-informed research priorities emerged as a useful method of strengthening research collaboration within and across continents. Partners from high-income countries primarily contributed methodological expertise; members of the cross-national research teams complemented one another in terms of content, context and methodological expertise and resources as well as research infrastructure. During the implementation phase, we will expand on this collaboration in an effort to build long-term capacity and infrastructure for evidence-based health care and public health in sub-Saharan Africa.

## References

[1] GBD 2013 Mortality and Causes of Death Collaborators. Global, regional, and national age-sex specific all-cause and cause-specific mortality for 240 causes of death, 1990–2013: a systematic analysis for the global burden of disease study 2013. Lancet. 2015 1 10;385(9963):117–71. 25530442 10.1016/S0140-6736(14)61682-2 PMC4340604

[2] Dussault G, Franceschini MC. Not enough there, too many here: understanding geographical imbalances in the distribution of the health workforce. Hum Resour Health. 2006;4(1):12. 10.1186/1478-4491-4-12 16729892 PMC1481612

[3] Ensor T, Cooper S. Overcoming barriers to health service access: influencing the demand side. Health Policy Plan. 2004 3;19(2):69–79. 10.1093/heapol/czh009 14982885

[4] Mayosi BM, Lawn JE, van Niekerk A, Bradshaw D, Abdool Karim SS, Coovadia HM; Lancet South Africa team. Health in South Africa: changes and challenges since 2009. Lancet. 2012 12 8;380(9858):2029–43. 10.1016/S0140-6736(12)61814-5 23201214

[5] Uthman OA, Wiysonge CS, Ota MO, Nicol M, Hussey GD, Ndumbe PM, et al. Increasing the value of health research in the WHO African Region beyond 2015–reflecting on the past, celebrating the present and building the future: a bibliometric analysis. BMJ Open. 2015;5(3):e006340. 10.1136/bmjopen-2014-006340 25770227 PMC4360830

[6] Health Ministers endorse a research strategy for the African Region. N’Djamena: World Health Organization Regional Office for Africa; 2015. Available from: http://www.afro.who.int/en/media-centre/pressreleases/item/8181-health-ministers-endorse-a-research-strategy-for-the-african-region.html [cited 2015 Dec 4].

[7] Chalmers I, Bracken MB, Djulbegovic B, Garattini S, Grant J, Gülmezoglu AM, et al. How to increase value and reduce waste when research priorities are set. Lancet. 2014 1 11;383(9912):156–65. 10.1016/S0140-6736(13)62229-1 24411644

[8] Ioannidis JPA, Greenland S, Hlatky MA, Khoury MJ, Macleod MR, Moher D, et al. Increasing value and reducing waste in research design, conduct, and analysis. Lancet. 2014 1 11;383(9912):166–75. 10.1016/S0140-6736(13)62227-8 24411645 PMC4697939

[9] Macleod MR, Michie S, Roberts I, Dirnagl U, Chalmers I, Ioannidis JPA, et al. Biomedical research: increasing value, reducing waste. Lancet. 2014 1 11;383(9912):101–4. 10.1016/S0140-6736(13)62329-6 24411643

[10] Sackett DL, Rosenberg WM, Gray JA, Haynes RB, Richardson WS. Evidence based medicine: what it is and what it isn’t. BMJ. 1996 1 13;312(7023):71–2. 10.1136/bmj.312.7023.71 8555924 PMC2349778

[11] Evidence-based methodologies for public health. Stockholm: European Centre for Disease prevention and Control; 2011.

[12] Birbeck GL, Wiysonge CS, Mills EJ, Frenk JJ, Zhou XN, Jha P. Global health: the importance of evidence-based medicine. BMC Med. 2013;11(223):223. 24228722 10.1186/1741-7015-11-223 PMC4190636

[13] Forland F, Rehfuess E, Klatser P, Kyamanywa P, Mayanja-Kizza H. Why evidence based approaches are urgently needed in Africa. Z Evid Fortbild Qual Gesundhwes. 2014;108(10):606–8. 10.1016/j.zefq.2014.10.025 25499116

[14] Lim SS, Vos T, Flaxman AD, Danaei G, Shibuya K, Adair-Rohani H, et al. A comparative risk assessment of burden of disease and injury attributable to 67 risk factors and risk factor clusters in 21 regions, 1990–2010: a systematic analysis for the global burden of disease study 2010. Lancet. 2012 12 15;380(9859):2224–60. 10.1016/S0140-6736(12)61766-8 23245609 PMC4156511

[15] Viergever RF, Olifson S, Ghaffar A, Terry RF. A checklist for health research priority-setting: nine common themes of good practice. Health Res Policy Syst. 2010;8:36. 21159163 10.1186/1478-4505-8-36 PMC3018439

[16] Doyle J, Waters E, Yach D, McQueen D, De Francisco A, Stewart T, et al. Global priority-setting for Cochrane systematic reviews of health promotion and public health research. J Epidemiol Community Health. 2005 3;59(3):193–7. 10.1136/jech.2003.019547 15709077 PMC1733031

[17] Schmucker C, Motschall E, Antes G, Meerpohl JJ. [Methods of evidence mapping. A systematic review]. Bundesgesundheitsblatt Gesundheitsforschung Gesundheitsschutz. 2013 10;56(10):1390–7. German. 10.1007/s00103-013-1818-y 23978984

[18] Rohwer A, Booth A, Pfadenhauer L, Brereton L, Gerhardus A, Mozygemba K, et al. Guidance on the use of logic models in health technology assessments of complex interventions. Integrate-HTA; 2016. Available from: http://www.integrate-hta.eu/downloads/ [cited 2016 Feb 9].

[19] Den Teufelskreis aus Armut und Krankheit durchbrechen. Press release 075/2015, 2 June 2015. Berlin: BMBF – German Ministry of Education and Research; 2015. Available from: https://www.bmbf.de/de/den-teufelskreis-aus-armut-und-krankheit-durchbrechen-91.html [cited 2015 June 11]. German.

[20] Bryant J, Sanson-Fisher R, Walsh J, Stewart J. Health research priority-setting in selected high income countries: a narrative review of methods used and recommendations for future practice. Cost Eff Resour Alloc. 2014;12(1):23. 10.1186/1478-7547-12-23 25873787 PMC4396165

[21] Oxman AD, Schünemann HJ, Fretheim A. Improving the use of research evidence in guideline development: 2. Priority-setting. Health Res Policy Syst. 2006;4(1):14. 10.1186/1478-4505-4-14 17134481 PMC1702532

[22] Lavis JN, Robertson D, Woodside JM, McLeod CB, Abelson J; Knowledge Transfer Study Group. How can research organizations more effectively transfer research knowledge to decision makers? Milbank Q. 2003;81(2):221–48, 171–2. 10.1111/1468-0009.t01-1-00052 12841049 PMC2690219

[23] Lomas J, Fulop N, Gagnon D, Allen P. On being a good listener: setting priorities for applied health services research. Milbank Q. 2003;81(3):363–88. 10.1111/1468-0009.t01-1-00060 12941000 PMC2690239

